# Generalized Drivers in the Mammalian Endangerment Process

**DOI:** 10.1371/journal.pone.0090292

**Published:** 2014-02-26

**Authors:** Manuela González-Suárez, Eloy Revilla

**Affiliations:** Department of Conservation Biology, Estación Biológica de Doñana (EBD-CSIC) Calle Américo Vespucio s/n, Sevilla, Spain; Bangor University, United Kingdom

## Abstract

An important challenge for conservation today is to understand the endangerment process and identify any generalized patterns in how threats occur and aggregate across taxa. Here we use a global database describing main current external threats in mammals to evaluate the prevalence of distinct threatening processes, primarily of anthropogenic origin, and to identify generalized drivers of extinction and their association with vulnerability status and intrinsic species' traits. We detect several primary threat combinations that are generally associated with distinct species. In particular, large and widely distributed mammals are affected by combinations of direct exploitation and threats associated with increasing landscape modification that go from logging to intense human land-use. Meanwhile, small, narrowly distributed species are affected by intensifying levels of landscape modification but are not directly exploited. In general more vulnerable species are affected by a greater number of threats, suggesting increased extinction risk is associated with the accumulation of external threats. Overall, our findings show that endangerment in mammals is strongly associated with increasing habitat loss and degradation caused by human land-use intensification. For large and widely distributed mammals there is the additional risk of being hunted.

## Introduction

Today, as anthropogenic degradation of the world's ecosystems is causing the Earth's sixth mass extinction event [1], it is becoming increasingly critical to understand the process of endangerment, how and why species become vulnerable to extinction and eventually disappear, to yield insight into how to prevent such extinctions. The extinction process has been studied from a theoretical and demographic perspective showing how population dynamics change as extinction approaches [2], [3]. However, findings from theoretical studies are sometimes difficult to translate into practical applications for management and conservation efforts. An alternative approach is to study the patterns by which external factors drive a species towards extinction. Because external threats can often be managed, a greater understanding of how threats combine and aggregate could guide management and conservation practice.

The process of endangerment can start with a direct perturbation or threat to a species, which is then followed by a suite of secondary factors that may act additively or synergistically to cause final extinction [4], [5]. Because the impact of a single threat is generally smaller than the cumulative impact of multiple threats [6], [7], as species become exposed to more threats, their risk of extinction often increases. Although in some cases a single threat may cause the extinction of a species, as was apparently the case for the Caribean monk seal [8], [9]. How threats occur and aggregate may be species- and context-dependent [10], [11], but there may also be interesting generalities within taxa that remain to be explored. Analyses of the mammalian threats described by the International Union for Conservation of Nature, IUCN [12], have shown that most species are affected by habitat loss and harvesting, whereas other threats are relatively infrequent [13]–[16]. However, these studies only considered presence vs. absence of threats, yet species are often affected by multiple threats that may interact. For example, habitat loss and harvesting may be widespread threats that affect species initially, reducing population size and habitat, while other factors, such as invasive species or disease, may provide the “coup de grace” that leads to extinction [17].

In this study we search for evidence of generalized drivers of extinction in mammals, exploring the prevalence and aggregation patterns of multiple external threats and the relationship between threat aggregation and vulnerability status. The IUCN and the Conservation Measures Partnership have generated a threats classification scheme [18] which identifies 11 main threat types that describe human actions and natural events that affect biodiversity (Table 1). Although these main types are subdivided into additional subcategories here we focused on the main types to reduce the idiosyncrasy associated with threat evaluations [19]. We use the listed threats to evaluate the prevalence and aggregation of extinction drivers considering also the patterns across distinct vulnerability status described by the IUCN Red List [12]. Because species' traits are known to influence species' vulnerability to certain threats [16], [20], we predict that there could be distinct threat patterns affecting different groups of species. In particular, ecologically specialized species are expected to be most affected by threatening processes like habitat loss and fragmentation; whereas species with slow reproductive life cycles should suffer most from threats that directly affect survival and fecundity such as direct exploitation or invasive species [11], [16], [20]–[22]. Therefore, we expect to find two general patterns, one characterized by threats that cause habitat loss and degradation that affects primarily habitat specialists with narrow distribution ranges, while large, slowly reproducing species are expected to be affected by direct exploitation. Overall our results show that certain threats combinations are much more frequent than others and as predicted, we identify two generalized patterns of threat aggregation that affect distinct groups of species.

**Table 1:** The 11 main categories defined by the IUCN threat classification scheme version 3.0 with the number of mammals affected by each in parenthesis.

*IUCN main threat category*	*Threat effect*	*Acronym*
1. Residential and commercial development (859)	Habitat: intense human use	*intense hab use* (I)
2. Agriculture and aquaculture (1613)	Habitat: agriculture	*agriculture* (A)
3. Energy production and mining (242)	Habitat: intense human use	*intense hab use* (I)
4. Transportation and service corridors (256)	Habitat: linear fragmentation	*fragmentation* (F)
5. Biological resource use (1906)		
5.1 Hunting & collecting of terrestrial animals (1049)	Direct exploitation	*exploitation* (E)
5.2 Gathering terrestrial plants (30)	Habitat: quality loss	*quality loss* (Q)
5.3 Logging and wood harvesting (1302)	Habitat: logging	*logging* (L)
5.4 Fishing and harvesting aquatic resources (143)	Direct exploitation	*exploitation* (E)
6. Human intrusions and disturbance (305)	Habitat: quality loss	*quality loss* (Q)
7. Natural system modifications (441)	Habitat: quality loss	*quality loss* (Q)
8. Invasive & other problematic species & genes (461)	Community disruption	*comm disruption* (C)
9. Pollution (187)	Habitat: quality loss	*quality loss* (Q)
10. Geological events (20)	Natural events	*natural* (N)
11. Climate change and severe weather (175)	Natural events	*natural* (N)

For all our analyses we reclassified the IUCN categories into eight threat effects that describe the ecosystem and species consequences of the listed human and natural actions.

## Methods

The IUCN threat classification scheme version 3.0 identifies ongoing threats for 2551 mammalian species, whereas the remaining 2940 have no listed threats under this classification scheme. The IUCN defines threats based on the human activity or natural event that induces a risk. However, similar actions may affect species differently. For example, the main threat type *biological resource use* includes both logging and hunting activities (Table 1). For mammals, the impact of being hunted, which leads to direct mortality, is clearly different from the effect of logging, which reduces available habitat (potentially affecting movement, reproduction and survival over time). Therefore, we reclassified the main IUCN threat types into eight threat effects (hereafter threats) that simplify the scheme and describe the consequences rather than the human actions (Table 1). Habitat modifications are classified into several categories that describe the intensity of the change [23] ranging from (generally) less intense changes such as logging and agriculture to more severe changes such as urban development. Our reclassification also simplifies the classification scheme to focus on main impacts, thus limiting the potential effects of subjectivity in threat assignment for minor detailed categories [19].

### Vulnerability to extinction and the number of threats

Because the number of listed threats varies across species we investigated the relationship between total number of threats and vulnerability status. The IUCN Red List has developed a ranking system that assesses conservation status of diverse taxa (Table S1, [12]). We tested if Red List status (an ordinal variable) increases with the number of listed ongoing threats in mammals controlling for knowledge, or data availability, for each species. We controlled for data availability as a surrogate of heterogeneous research effort on different species because our understanding of the threatening processes is likely greater for well-studied species, thus well-studied species are more likely to have more threats listed. In addition, we have previously shown [24] that fewer data are available for threatened mammals, thus data availability could be a confounding factor. Data availability was independently defined by the (log_10_-transformed) number of studies per species used to populate the PanTHERIA database of mammalian trait data (methods described in [24]). We followed the IUCN taxonomy and matched names to PanTHERIA's taxonomy using the synonyms listed by the IUCN when necessary.

To address the issue that related species are not independent observations [25] we used taxonomically-corrected generalized linear mixed models, GLMM [26] that include order, family and genus as nested random effects. Models were fitted using the ‘GLIMMIX’ procedure in sas 9.2 (SAS Institute Inc., Cary, NC, USA) with a multinomial distribution (cumulative logit link). We used taxonomic correction rather than phylogenetic correction because to our knowledge, there are not frequentist phylogenetic ordinal regression approaches currently available. The Red List uses five criteria to define status based on population reduction, distribution range size, population size and decline, and small or restricted populations. Species in naturally small fragmented ranges may be defined as threatened under criterion B because they are vulnerable to extinction even if they are affected by few or no extinction drivers. Therefore, we tested the relationship between Red List status and number of threats for all species and also for a subset that excludes those listed under criterion B. Data deficient species were excluded because of their unknown vulnerability status. Extinct and extinct in the wild species were not considered because, by definition, they have no listed ongoing threats.

For GLMM analyses we report the estimated regression coefficient (b), its standard error (SE), and the *P*-value of a likelihood ratio test comparing models with and without the fixed factor (but including taxonomic random effects). We also report the conditional deviance R^2^_D(c)_ and marginal deviance R^2^_D(m)_ calculated as 1-(full model deviance)/(null model deviance). The full model was the model including fixed and random factors. R^2^_D(c)_ was calculated with a null model that includes random factors and thus, describes the improvement in model fit due to the fixed factor(s) alone. R^2^_D(m)_ was calculated with a null model that only includes an intercept, and thus describes the improvement in model fit due to both fixed and random factors.

### Threat prevalence and combinations

Using our threat reclassification we identified existing threat combinations among mammals and calculated their prevalence (how many species were affected by each threat and threat combination). We then selected the main combinations (combinations found in >10% of the species with the same number of listed threats) to explore if species affected by the same threats share common traits. In particular, we focused on two species' traits: distribution range area, which is generally associated with specialization [27], and adult body mass, which is associated with reproductive speed [28]. Both of these traits were estimated for many mammals [24] and have been repeatedly associated with vulnerability to extinction [10], [26], [29]. Body mass data were obtained from the database PanTHERIA [30]. Distribution range areas were calculated using the IUCN global distribution range map [12] and the cylindrical equal area projection. To better visualize how species traits associate with threat combinations we used a principal component analysis (PCA) summarizing adult body mass and range area for the 3435 species with available data on both traits. We applied the ‘princomp’ function in r [31] using the covariance matrix of adult body mass and range area. Both variables were log_10_-transformed and standardized by subtracting the mean and dividing by the standard deviation. The first component (PC1) extracted from the PCA explains 59% of the variance in adult body mass and range area, with high values indicating large, widely distributed species (PC1 loadings: range area = 0.35, adult body mass = 0.93). Differences in trait values among main threat combinations and group of species with different numbers of listed threats were tested using phylogenetic generalized least square (pgls) regression models. Pgls models control for the evolutionary relationships among species, thus addressing the issue that data from related species do not represent independent observations [25]. We used the ‘pgls’ function in the ‘caper’ package in r estimating lambda with the maximum likelihood method [31]. Phylogeny was represented by the updated mammalian supertree [32], [33]. For pgls results we report the estimated regression coefficient (b) and its standard error (SE) for continuous dependent variables and the *F* statistic for categorical dependent variables, in all cases we include the *P*-value and R^2^ estimated by ‘caper’.

Finally, we characterized the terrestrial distribution of the most common threat combinations using the IUCN distribution range maps (including only regions where presence was described as “Extant” or “Probably Extant” and origin listed as “Native”) and assuming threats are homogeneously distributed throughout the range [14]. Distribution maps were projected in the cylindrical equal area projection and onto a grid with cell area equal to 31,490 km^2^. The size of the grid was selected in accordance to the resolution of the available distribution maps [34]. For each grid cell we report the proportion of species with given combinations over the total of species in the grid and the mean number of threats per species in each combination group as an indication of the accumulated risk.

## Results

### Vulnerability to extinction and the number of threats

**Figure 1:**
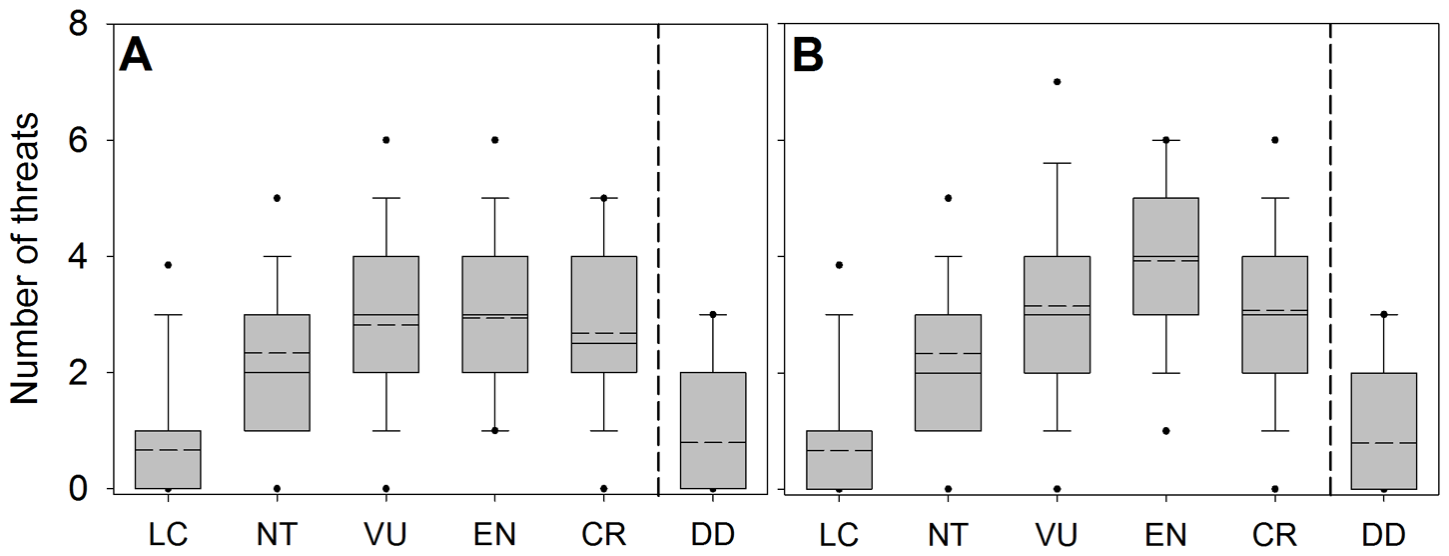
Number of ongoing threats per species for each IUCN Red List status (including species with no threats). A) all species, B) excluding species listed as threatened under criterion B (small range area). Boxes represented the 25–50% percentiles, with the median indicated by a solid line and the arithmetic mean by a dashed line. The whiskers show the 10 and 90% percentiles with symbols showing the 5 and 95% percentiles. LC = least concern, NT = near threatened, VU = vulnerable, EN = endangered, CR = critically endangered. Extinct and extinct in the wild species have no ongoing threats listed under the classification scheme and are not represented.

Vulnerability as defined by the IUCN Red List status tends to increase with the number of listed threats per species even controlling for the reduced data availability of more vulnerable species (GLMM R^2^_D(c)_ = 0.19, R^2^_D(m)_ = 0.24; number of threats b = 0.88, SE = 0.030, *P*<0.001; data availability b = −1.31, SE = 0.082, *P*<0.001. Fig. 1a). Overall, least concern species have the fewest number of listed threats, while endangered species have the most (Fig. 1a). When species listed under criterion B are excluded the number of listed threats increases more linearly with vulnerability (GLMM R^2^_D(c)_ = 0.17, R^2^_D(m)_ = 0.28; b = 0.82, SE = 0.035, *P*<0.001. Fig. 1b).

Listing of threats may be less reliable for least concern species because these species are not considered to be at risk and thus, less emphasis is placed on reporting their threats. Similarly, species without listed threats may not have been adequately evaluated and thus, lack of threats may in some cases not reflect true conditions [16], [19]. Therefore, the observed increase in listed threats with Red List status could be an artifact of the threat listing limitations. However, the pattern is consistent and the number of threats still increases with vulnerability to extinction after excluding least concern species (GLMM including species listed under criterion B, R^2^_D(c)_ = 0.07, R^2^_D(m)_ = 0.08; b = 0.20, SE = 0.041, *P*<0.001. GLMM excluding species listed under criterion B, R^2^_D(c)_ = 0.07, R^2^_D(m)_ = 0.13; b = 0.17, SE = 0.053, *P*<0.001) and after excluding species without listed threats (GLMM including species listed under criterion B, R^2^_D(c)_ = 0.08, R^2^_D(m)_ = 0.11; b = 0.33, SE = 0.037, *P*<0.001. GLMM excluding species listed under criterion B, R^2^_D(c)_ = 0.08, R^2^_D(m)_ = 0.15; b = 0.42, SE = 0.045, *P*<0.001).

In all cases we find that critically endangered species tend to have fewer threats than those listed as endangered. As a species approaches its demise few threats may remain relevant or be perceived as such by evaluators. In addition, critically endangered species have small ranges that may overlap with fewer human activities or may be more actively managed leading to a true reduction of anthropogenic threats. Although there are no listed ongoing threats for extinct species, some extinct species have past threats listed. Among the 48 extinct species with listed past threats, the median number of described threats is low ( = 1), probably because only the final culprits are identified. As expected, species listed as data deficient also have few listed threats because little is known about these species and additionally because evaluators are not required to list existing threats for data deficient species.

### Threat prevalence and combinations

**Figure 2:**
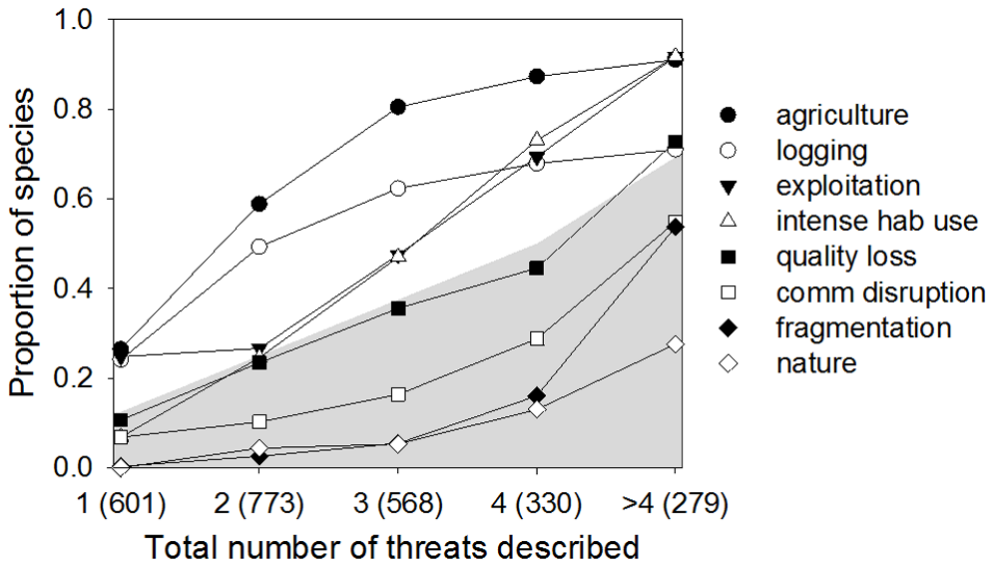
Threat prevalence for species with distinct numbers of listed threats (levels). The number of species in each level is indicated in parenthesis (species in all Red List status are included). Values that fall within the shaded area indicate fewer species than expected if all threats were equally likely (e.g., for species with one threat the expected proportion of species suffering from each threat is 1/8, for two threats is 2/8, etc. For >4 threats the proportion was calculated based on the mean number of threats = 5.6).

If all threats were equally likely to be listed, the expected proportion of species suffering from each threat when a single threat is listed would be on average 1/8, when two threats are listed the proportion would be 2/8, and so forth. Among species with a single threat we find three threats that affect more than 1/8 of the species: *agriculture, exploitation*, and *logging* (Fig. 2). These three threats remain common in all other levels (two or more listed threats). In addition, a fourth driver, *intense hab use*, affects more species than expected among those with two listed threats, while habitat *quality loss* becomes common among species with >4 threats. This pattern is consistent across species in different Red List status (Fig. S1). In addition to being more or less common, we also find that the proportion of species affected by some threats changes rapidly in some cases (for example the prevalence of *intense hab use* increases rapidly comparing species with three and four listed threats, Fig. 2) while other threats barely change their frequency in groups with different numbers of listed threats (e.g., *nature*).

**Figure 3:**
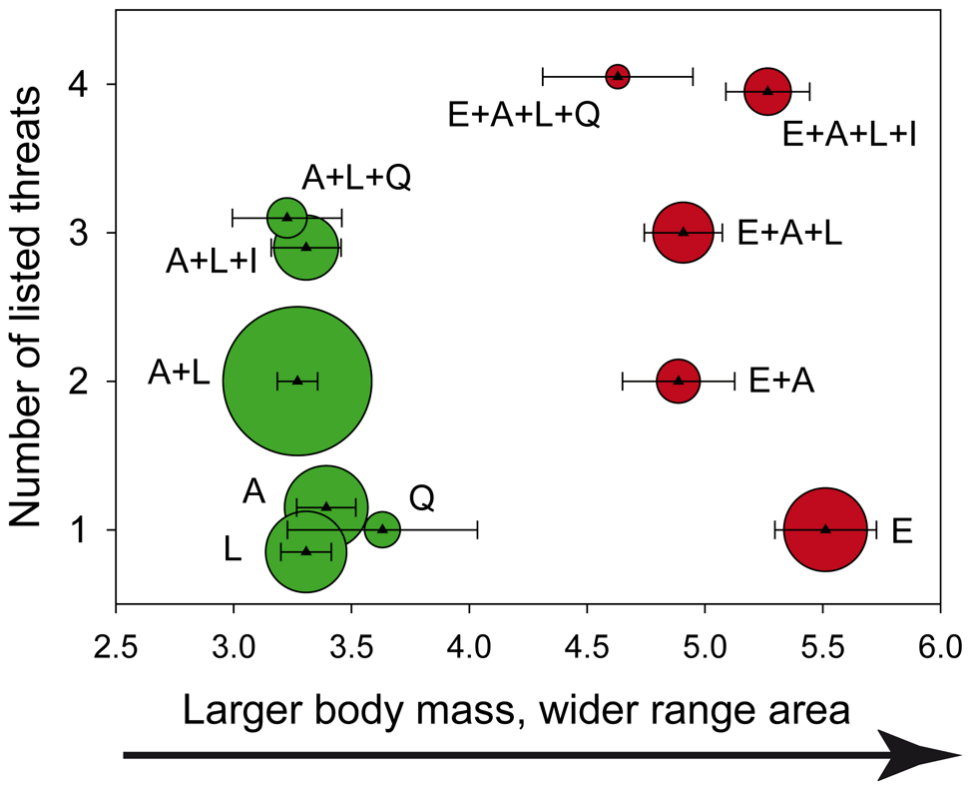
Main threat combinations observed among mammals with distinct numbers of threats. Each combination is represented by a colored circle with size proportional to the number of species with that combination. For each combination we also plot the mean (small triangle) and the standard error of the mean (error bars) of a PC component representing adult body masses and distribution range areas values. Combinations in the exploitation-habitat loss group are represented by red circles, while those in the habitat loss and degradation group are represented by green circles. Threats are described in Table 1: A =  Habitat: agriculture, L =  Habitat: logging, E =  Direct exploitation, I =  Habitat: intense human use, Q =  Habitat: quality loss.

To further describe existing threat combinations we focused on species with 1–4 listed threats. Few species have >4 listed threats and additionally this range matches the median number of threats of each of the Red List status going from least concern to endangered once species listed under criterion B are removed (Fig. 1b). We find diverse threat combinations occurring at varying frequencies, some being quite common while others only affect a few species (Tables S2–S5). Considering the most common combinations (affecting >10% of the species with the same number of threats) we identified two main groups of mammals that differ in their threats and traits (Fig. 3; see Fig. S2 for each trait separately). One group (habitat loss and degradation) includes exclusively threats associated with human land-use intensification that cause habitat loss and degradation, while the second group (exploitation-habitat loss) includes direct exploitation with the addition of habitat loss caused by land-use intensification. Species in the exploitation-habitat loss group are primarily large, widely distributed mammals (median body mass: 3,430 g, median range area: 267,600 km^2^) compared to those affected by combinations including only habitat loss and degradation (median body mass: 52.0 g, median range area: 34,850 km^2^). A phylogenetically-corrected model shows that these differences, as described by the first component of a principal component analysis, are statistically significant (*N* = 734, note that 17 species are not represented in the phylogeny and had to be excluded from the analyses; pgls *F* = 59.02, *P*<0.001, R^2^ = 0.07).

Among species in the exploitation-habitat loss group those with higher number of listed threats have smaller range areas (*N* = 421, 39 species are not represented in the phylogeny; pgls b = −0.01, SE = 0.005, *P* = 0.005, R^2^ = 0.02) suggesting that range contraction could occur as the number of threats increases (Fig. S2). Among species in the habitat loss and degradation group we find no significant changes in range size (*N* = 697, 122 species not represented in the phylogeny; pgls b = −0.01, SE = 0.007, *P* = 0.43, R^2^ = 0.00. Fig. S2). Adult body mass does not change with the number of listed threats in the exploitation-habitat loss group (*N* = 318, 1 species not represented in the phylogeny; pgls b = 0.00, SE = 0.003, *P* = 0.93, R^2^ = 0.00. Fig. S2) or the habitat loss and degradation group (*N* = 416, 16 species not represented in the phylogeny; pgls b = 0.00, SE = 0.006, *P* = 0.99, R^2^ = 0.00. Fig. S2).

**Figure 4:**
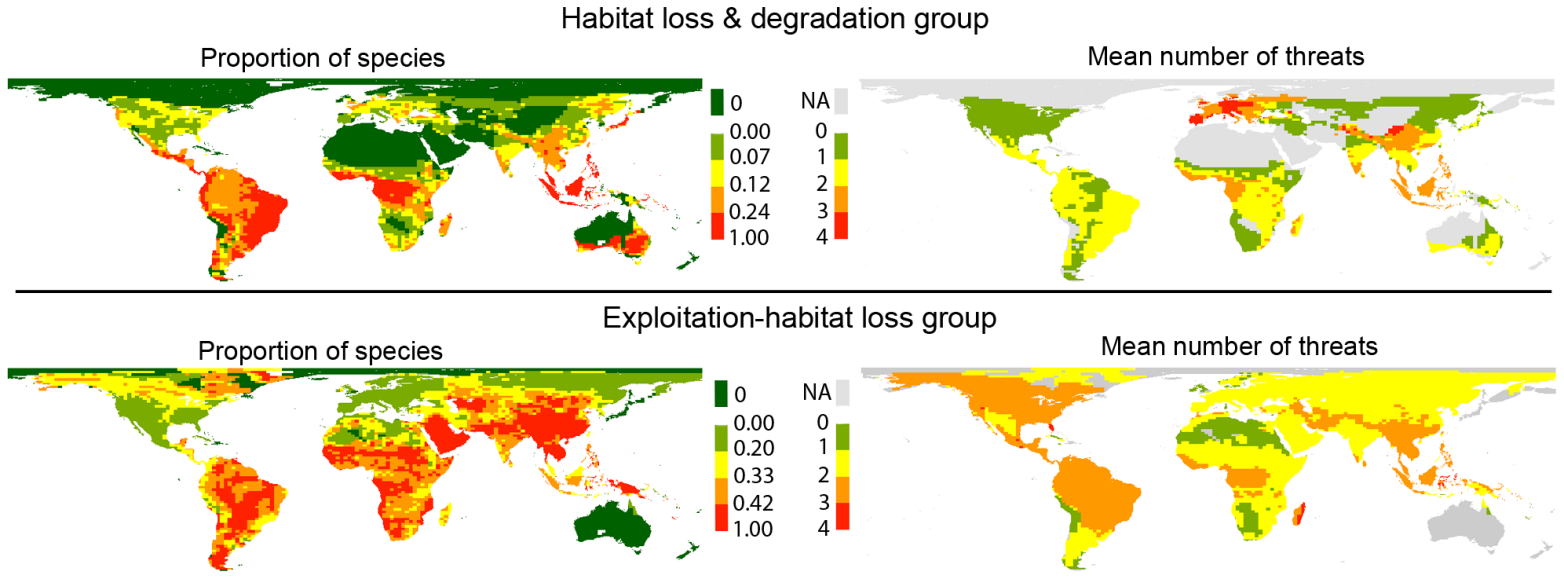
Spatial distribution of terrestrial mammals from the two main threat groups (described in Fig. 3). Left panels show the proportion of species in each group (calculated over the total of species with 1–4 listed threats). Right panels show the mean number of listed threats in each grid for the species in each group. *NA* indicates no species in the group found in that cell.

The spatial representations show that the majority of terrestrial mammals in the habitat loss and degradation group are found in tropical areas but these species have a relatively low mean number of threats in those areas (Fig. 4). Meanwhile, the mammals on the exploitation-habitat loss group are more widespread but have a greater mean number of listed threats in tropical areas (Fig. 4).

## Discussion

Using available data on the external threats affecting mammalian species worldwide we estimate prevalence and identify common threat combinations. We find that some threat combinations are much more likely than expected if all threats were equally probable to be listed while others rarely occurred. Small, narrowly-distributed mammals are affected by combinations of threats describing habitat loss and degradation resulting from human land-use intensification. Large, more widely distributed species are also affected by threats reflecting human land-use intensification leading to habitat loss and range contraction but have the additional risk of being exploited. In addition to the main groups some species have less common threat combinations which may reflect processes idiosyncratic to the organism and the human-environmental context. We find that while habitat modification affects both large and small mammals this threat may have potentially different consequences for each group [29], [33]. Distribution range sizes are smaller among the larger mammals with more listed threats within the exploitation-habitat loss group, but not among the small, already narrowly distributed species in the habitat loss and degradation group, even though these species are likely those most affected by habitat modifications [16]. This suggests that range contraction may occur among large, more widely distributed species as the number of threats affecting them increases.

Terrestrial mammals in the habitat loss and degradation group are found primarily in tropical areas which also have high mammalian richness [14] and the main remaining natural habitats [35]. Unfortunately, these are also regions undergoing fast human land-use intensification leading to increasing habitat loss and degradation [36]. Currently, the mean number of threats for species in the habitat loss and degradation group that live in these regions is relatively low. However, given the rate of habitat loss and land-use intensification in areas such as Amazonia and Southeast Asia the number of threats affecting these species may change quickly. The species with most threats in the habitat loss and degradation group are presently found in Europe and central Asia, areas which have already undergone intense habitat modification and degradation that may affect particularly these small, narrowly distributed species [33]. Species in the exploitation-habitat loss group are more widespread but, in contrast with the pattern described above, the mean number of listed threats for these mammals in tropical areas is high. This pattern may be explained by the fact that many exploitable mammals from Europe are long extinct [37] or currently protected and managed, and because of the high hunting pressure on larger mammals in tropical areas [33]. A previous study that represented the spatial distribution of threats [14] also reported that tropical areas have the highest density of species threatened by habitat loss. However, this study also identified Southeast Asia as a hotspot for harvested species, while in our analyses species in the exploitation-habitat loss group are much more widespread. The difference may be because Schipper et al. [14] represented the total number of species rather than the proportion, thus not correcting for the confounding effect of overall mammalian richness. In addition, Schipper et al. considered all species listed as affected by harvesting (which includes those affected by *exploitation* and *logging*) while we only represent those affected by *exploitation*.

A limitation of our analysis is that IUCN threats are currently not rated in importance and thus, some listed threats may pose a relatively minor risk but the IUCN listing and, by necessity, our analyses treat all listed threats as equal. Our focus on main threats and a simplified scheme partly reduced the spurious effects of considering rare threats but we agree with Hayward [19] on the need to standardize the threat assignment because understanding the actual causes of decline is essential for effective conservation. In addition, it would also be helpful, albeit challenging, to rate threat importance to further clarify how different drivers of extinction influence species at the different stages in the path to extinction [15]. Some threats, e.g., *comm disruption*, may be relatively rare but could pose a great risk because they primarily affect already vulnerable species and have direct, and possibly devastating, effects on individual survival [17]. As a first step towards including a rating system, instructions accompanying an update to the 3.0 version released on June 2012 [38] recommend recording the scope and severity of the threats. We encourage the publication of the resulting ranking of threats.

An interesting hypothesis derived from our results is the possibility of inferring temporal patterns of threat accumulation associated with increased vulnerability to extinction, with two main paths affecting distinct species. If we consider the endangerment process as an accumulation of threats over time, the IUCN listing could be considering as a snapshot of this process that offers insights into how threats may accumulate defining the path to extinction. The path may appear to start with general threats such as *logging* or *exploitation* to which threats involving more intense habitat modifications are added. This possible sequence corresponds very well with the temporal changes in land-use intensity described by Foley et al. [23]. These authors propose that initially the land is in its natural state with humans using available resources (hunting and gathering) and possibly starting initial frontiers clearing (logging). Therefore, species would initially be affected by processes like *exploitation* (particularly prevalent among large, widely distributed species) and *logging* (more common among small, more narrowly distributed species, possibly specialists [27]). Subsequently Foley et al. [23] suggest the land is modified by subsistence agriculture and small-scale farms, which are followed by additional habitat modification generated by an intensification of agriculture and urbanization. Our results suggest that indeed threats associated with land-use intensification affect all species, with less intense threats being more common for species with fewer listed threats.

The existence of such temporal paths to extinction describing the accumulation (and intensification) of threats could be tested using temporal data on threats appearance, which to our knowledge are not currently available at a global scale. However, such temporal information could become available in the future if the IUCN reassesses the presence of ongoing threats for mammals using the same (or equivalent) threat classification system. The evaluations completed up to date have used different classification schemes limiting the use of these data to make temporal inferences. Nevertheless, our results offer some support to the interesting hypothesis that threats accumulate and intensify over time increasing extinction risk with different types of species experiencing different threat combinations and accumulation patterns. Whether these patterns truly reflect a temporal sequence and are also observable in other taxa remains to be explored.

For conservation practice our results at a global scale support the importance of first preserving natural habitats and then halting and reversing human land-use intensification. Intensifying habitat degradation is generally associated with higher vulnerability and its effects appear to be cumulative. In particular, there is a need to protect habitat in tropical areas where many small, narrowly distributed species occur but still have relatively low numbers of threats; thus these species may be effectively protected simply by preserving their remaining suitable habitat. Importantly, our results suggest that halting the land-use intensification process at any stage could be a good precautionary approach even in degraded areas because apparently greater intensification increases the risk by generating additional threats. Despite these general conclusions, particular actions should be defined after careful analysis of the threats affecting a given species locally, as well as evaluating the plausible measures that may be taken to stop or mitigate such threats. In certain regions, different threat patterns may be prevalent because globally rare threats are locally common. For example, invasive species are a serious problem in many islands [39] even if relatively rare at a global scale. In addition, some threats such as climate change may become much more relevant in the future, contributing to changes in habitat and communities [40]. Threats that are currently relatively infrequent could become the final factors that drive species to extinction once the most widespread sources of risk (*agriculture*, *logging* and *exploitation*) are in effect [17]. Because we live in a humanized planet in which initial drivers may soon be ubiquitous [41], [42] these additional, now rare, threats are likely to become more common in the future.

## Supporting Information

Figure S1 **Frequency of each threat effect among species with different number of listed threats.** Each panel represents species in each Red List status: LC (Least Concern), NT (Near Threatened), VU (Vulnerable), EN (Endangered), CR (Critically Endangered), DD (Data Deficient). Numbers in parenthesis indicate the number of species. Values that fall within the shaded area indicate fewer species than expected if all threats were equally likely (e.g., for species with one threat the expected proportion of species suffering from each threat is 1/8, for two threats is 2/8, etc. For >4 threats the proportion was calculated based on the average number of threats in each group).
(TIF)

Figure S2 **Main threat combinations observed among mammals with distinct numbers of threats.** Each combination is represented by a colored circle with size proportional to the number of species in that combination. For each combination we also plot the mean (small triangle) and the standard error of the mean (error bars) of the adult body masses (left panel) and the distribution range areas (right panel) in the group. Red circles represent combinations we assigned to an exploitation-habitat loss group, and green circles combinations assigned to a habitat loss and degradation group. Threats are described in table 1: A =  Habitat: agriculture, L =  Habitat: logging, E =  Direct exploitation, I =  Habitat: intense human use, Q =  Habitat: quality loss.
(TIF)

Table S1 **Description of the Red List status categories defined by the IUCN and used in the analyses.**
(DOCX)

Table S2 **Observed threat combinations for mammals with one listed threat.**
(DOCX)

Table S3 **Observed threat combinations for mammals with two listed threats.**
(DOCX)

Table S4 **Observed threat combinations for mammals with three listed threats.**
(DOCX)

Table S5 **Observed threat combinations for mammals with four listed threats.**
(DOCX)
