# Supplementary figures and images for: Generalized Drivers in the Mammalian Endangerment Process
Source: PLoS One. 2014 Feb 26;9(2):e90292. doi: 10.1371/journal.pone.0090292 (PMC3936011; doi:10.1371/journal.pone.0090292)

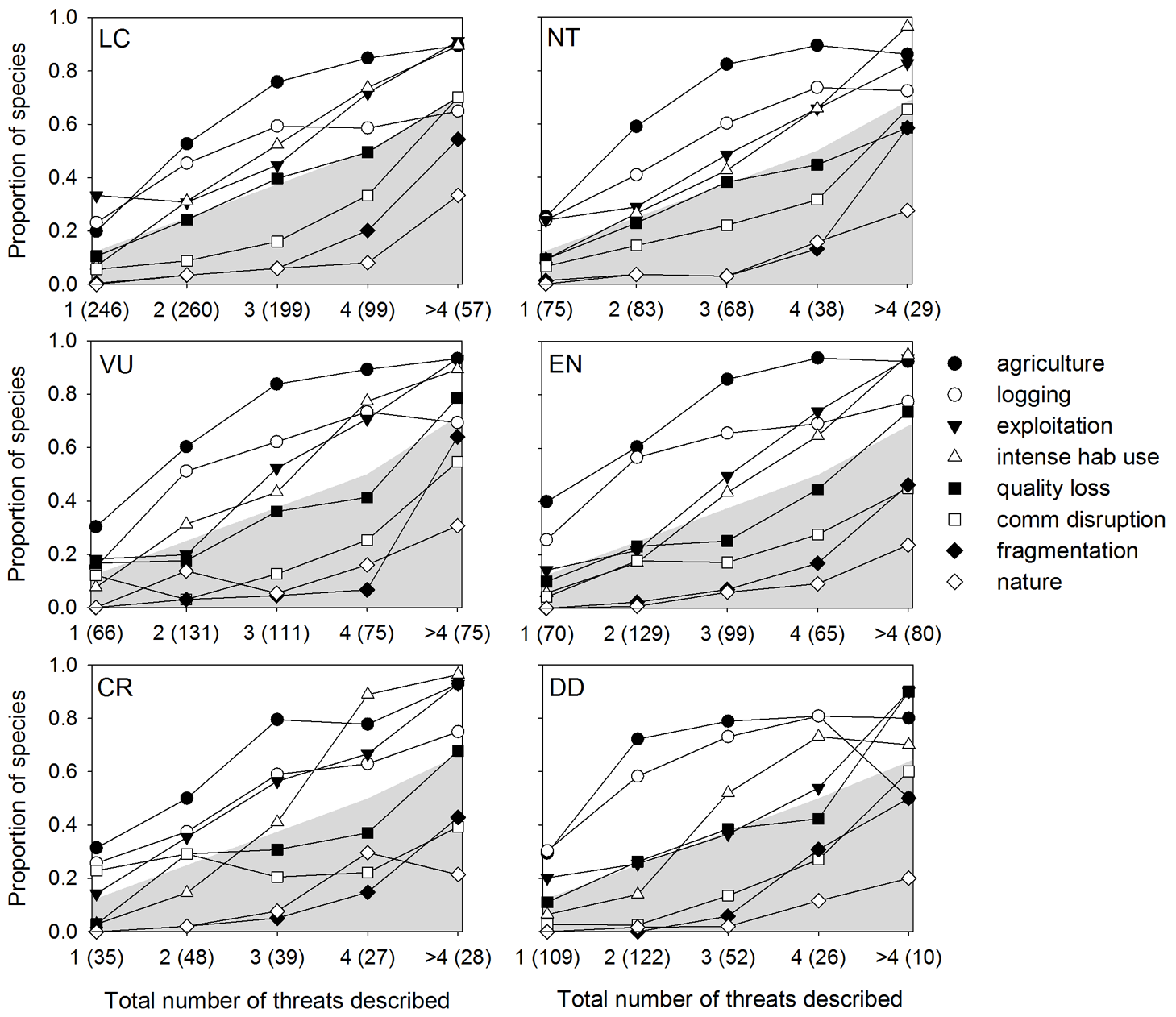

Supplement: Figure S1 — Frequency of each threat effect among species with different number of listed threats. Each panel represents species in each Red List status: LC (Least Concern), NT (Near Threatened), VU (Vulnerable), EN (Endangered), CR (Critically Endangered), DD (Data Deficient). Numbers in parenthesis indicate the number of species. Values that fall within the shaded area indicate fewer species than expected if all threats were equally likely (e.g., for species with one threat the expected proportion of species suffering from each threat is 1/8, for two threats is 2/8, etc. For >4 threats the proportion was calculated based on the average number of threats in each group). (TIF) [file pone.0090292.s001.tif]

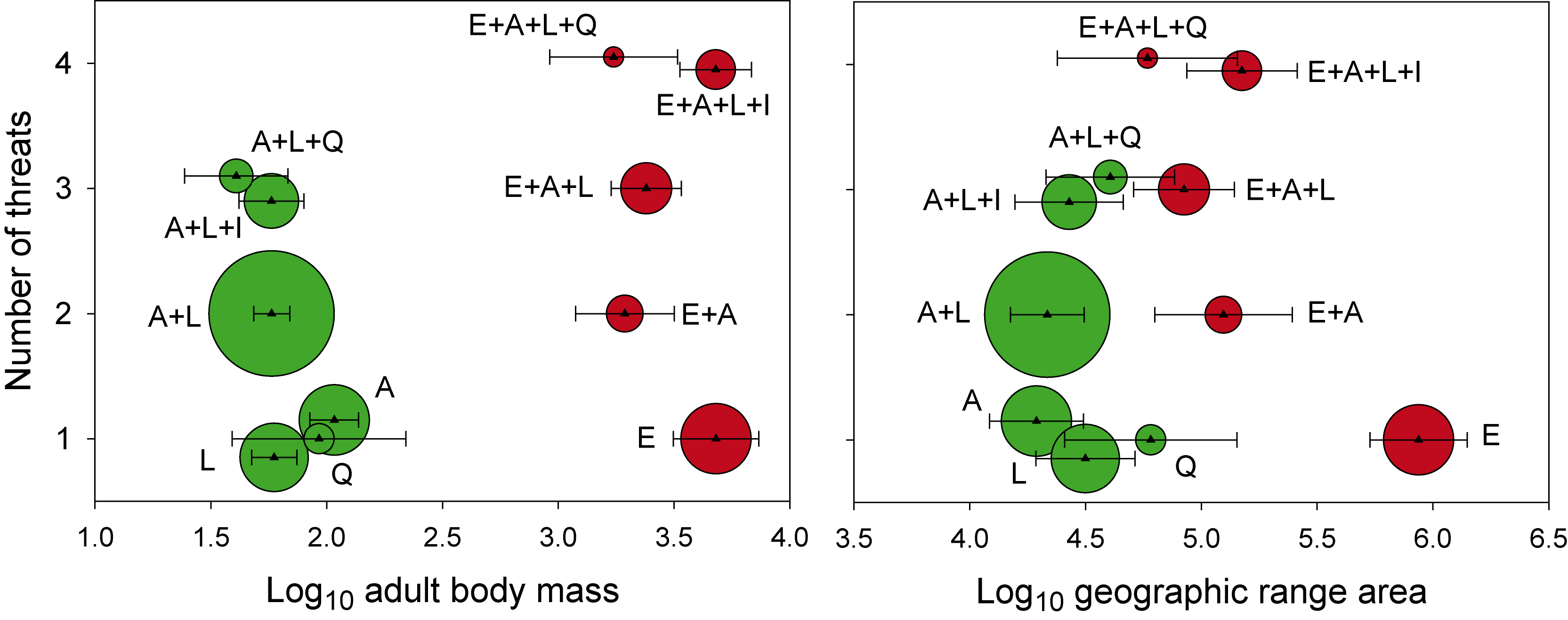

Supplement: Figure S2 — Main threat combinations observed among mammals with distinct numbers of threats. Each combination is represented by a colored circle with size proportional to the number of species in that combination. For each combination we also plot the mean (small triangle) and the standard error of the mean (error bars) of the adult body masses (left panel) and the distribution range areas (right panel) in the group. Red circles represent combinations we assigned to an exploitation-habitat loss group, and green circles combinations assigned to a habitat loss and degradation group. Threats are described in table 1: A = Habitat: agriculture, L = Habitat: logging, E = Direct exploitation, I = Habitat: intense human use, Q = Habitat: quality loss. (TIF) [file pone.0090292.s002.tif]
