# Supplementary material for: Generalized Drivers in the Mammalian Endangerment Process
Source: PLoS One. 2014 Feb 26;9(2):e90292. doi: 10.1371/journal.pone.0090292 (PMC3936011; doi:10.1371/journal.pone.0090292)
Supplement: Table S1 — Description of the Red List status categories defined by the IUCN and used in the analyses. (DOCX) [file pone.0090292.s003.docx]

**Table S1.** Description of the Red List status categories defined by the IUCN and used in the analyses.

| Category | Definition |
| --- | --- |
| Extinct | No reasonable doubt that the last individual has died. |
| Extinct in the Wild | Known only to survive in cultivation, in captivity or as a naturalized population (or populations) well outside the past range. |
| Critically Endangered | Extremely high risk of extinction in the wild. |
| Endangered | Facing a very high risk of extinction in the wild. |
| Vulnerable | Facing a high risk of extinction in the wild. |
| Near Threatened | Close to qualifying for or is likely to qualify for a threatened category in the near future. |
| Least Concern | Widespread and abundant. |
| Data Deficient | Inadequate information to make an assessment. |

Threatened species are those in the Critically Endangered, Endangered o Vulnerable categories. A full description of the categories and classification criteria can be downloaded from (http://www.iucnredlist.org/technical-documents/categories-and-criteria).
