# Supplementary material for: Generalized Drivers in the Mammalian Endangerment Process
Source: PLoS One. 2014 Feb 26;9(2):e90292. doi: 10.1371/journal.pone.0090292 (PMC3936011; doi:10.1371/journal.pone.0090292)
Supplement: Table S2 — Observed threat combinations for mammals with one listed threat. (DOCX) [file pone.0090292.s004.docx]

**Table S2.** Observed threat combinations for mammals with one listed threat.

| A | E | L | Q | I | C | F | Na | *N* | % |
| --- | --- | --- | --- | --- | --- | --- | --- | --- | --- |
| **x** |  |  |  |  |  |  |  | **159** | **26.5** |
|  | **x** |  |  |  |  |  |  | **149** | **24.8** |
|  |  | **x** |  |  |  |  |  | **145** | **24.1** |
|  |  |  | **x** |  |  |  |  | **64** | **10.6** |
|  |  |  |  | x |  |  |  | 41 | 6.8 |
|  |  |  |  |  | x |  |  | 41 | 6.8 |
|  |  |  |  |  |  | x |  | 2 | 0.3 |

We list all observed combinations indicating the threats included (A=*agriculture*, L=*logging*, E=*exploitation*, I=*intense hab use*, Q=*quality* *loss*, C=*comm disruption*, F=*fragmentation*, and Na=*nature*); the number of species with that combination (*N*) and the percentage (%) they represent from all species with the same level (one listed threat). Note that no species have a single listed threat corresponding to *nature* (Na). Combinations represented in the main text figure 3 and supplementary figure S2 are in bold.
