# Supplementary material for: Generalized Drivers in the Mammalian Endangerment Process
Source: PLoS One. 2014 Feb 26;9(2):e90292. doi: 10.1371/journal.pone.0090292 (PMC3936011; doi:10.1371/journal.pone.0090292)
Supplement: Table S3 — Observed threat combinations for mammals with two listed threats. (DOCX) [file pone.0090292.s005.docx]

**Table S3.** Observed threat combinations for mammals with two listed threats.

| A | E | L | Q | I | C | F | Na | *N* | % |
| --- | --- | --- | --- | --- | --- | --- | --- | --- | --- |
| **x** |  | **x** |  |  |  |  |  | **266** | **34.4** |
| **x** | **x** |  |  |  |  |  |  | **78** | **10.1** |
| x |  |  |  | x |  |  |  | 55 | 7.1 |
|  |  |  | x | x |  |  |  | 51 | 6.6 |
|  | x |  | x |  |  |  |  | 42 | 5.4 |
| x |  |  | x |  |  |  |  | 42 | 5.4 |
|  | x | x |  |  |  |  |  | 41 | 5.3 |
|  |  |  |  | x |  |  | x | 34 | 4.4 |
|  |  | x |  | x |  |  |  | 27 | 3.5 |
|  |  | x | x |  |  |  |  | 23 | 3.0 |
|  |  | x |  |  | x |  |  | 23 | 3.0 |
|  |  |  | x |  | x |  |  | 21 | 2.7 |
|  | x |  |  | x |  |  |  | 17 | 2.2 |
|  | x |  |  |  | x |  |  | 17 | 2.2 |
| x |  |  |  |  | x |  |  | 12 | 1.6 |
|  | x |  |  |  |  | x |  | 11 | 1.4 |
|  |  |  |  | x | x |  |  | 4 | 0.5 |
| x |  |  |  |  |  | x |  | 2 | 0.3 |
|  |  |  |  | x |  | x |  | 2 | 0.3 |
|  |  |  | x |  |  | x |  | 2 | 0.3 |
|  |  |  |  |  | x | x |  | 2 | 0.3 |
|  |  | x |  |  |  | x |  | 1 | 0.1 |

We list all observed combinations indicating the threats included (A=*agriculture*, L=*logging*, E=*exploitation*, I=*intense hab use*, Q=*quality* *loss*, C=*comm disruption*, F=*fragmentation*, and Na=*nature*); the number of species with that combination (*N*) and the percentage (%) they represent from all species with two listed threats. Combinations represented in the main text figure 3 and supplementary figure S2 are in bold.
