# Supplementary material for: Generalized Drivers in the Mammalian Endangerment Process
Source: PLoS One. 2014 Feb 26;9(2):e90292. doi: 10.1371/journal.pone.0090292 (PMC3936011; doi:10.1371/journal.pone.0090292)
Supplement: Table S4 — Observed threat combinations for mammals with three listed threats. (DOCX) [file pone.0090292.s006.docx]

**Table S4.** Observed threat combinations for mammals with three listed threats.

| A | E | L | Q | I | C | F | Na | *N* | % |
| --- | --- | --- | --- | --- | --- | --- | --- | --- | --- |
| **x** |  | **x** |  | **x** |  |  |  | **116** | **20.4** |
| **x** | **x** | **x** |  |  |  |  |  | **109** | **19.2** |
| **x** |  | **x** | **x** |  |  |  |  | **71** | **12.5** |
| x | x |  |  | x |  |  |  | 47 | 8.3 |
|  | x |  | x |  | x |  |  | 23 | 4.0 |
| x |  |  | x |  | x |  |  | 19 | 3.3 |
| x | x |  | x |  |  |  |  | 18 | 3.2 |
| x | x |  |  |  | x |  |  | 18 | 3.2 |
| x |  |  | x | x |  |  |  | 17 | 3.0 |
|  | x |  | x | x |  |  |  | 15 | 2.6 |
|  | x | x |  | x |  |  |  | 13 | 2.3 |
| x |  |  |  | x |  |  | x | 13 | 2.3 |
| x |  | x |  |  | x |  |  | 10 | 1.8 |
|  |  |  | x | x |  |  | x | 9 | 1.6 |
| x |  |  |  | x |  | x |  | 8 | 1.4 |
|  | x | x | x |  |  |  |  | 8 | 1.4 |
|  |  | x | x | x |  |  |  | 8 | 1.4 |
| x |  | x |  |  |  | x |  | 6 | 1.1 |
| x | x |  |  |  |  | x |  | 4 | 0.7 |
|  |  | x | x |  | x |  |  | 4 | 0.7 |
|  |  |  | x | x | x |  |  | 4 | 0.7 |
|  | x | x |  |  | x |  |  | 3 | 0.5 |
|  | x |  |  | x | x |  |  | 3 | 0.5 |
|  | x |  |  |  | x | x |  | 3 | 0.5 |
|  |  |  |  | x | x | x |  | 3 | 0.5 |
|  | x |  |  | x |  |  | x | 3 | 0.5 |
|  |  | x |  | x |  |  | x | 3 | 0.5 |
|  | x |  | x |  |  | x |  | 2 | 0.4 |
|  |  | x | x |  |  | x |  | 2 | 0.4 |
|  |  |  | x | x |  | x |  | 2 | 0.4 |
|  |  |  |  | x | x |  | x | 2 | 0.4 |
|  | x | x |  |  |  | x |  | 1 | 0.2 |
| x |  |  |  | x | x |  |  | 1 | 0.2 |

We list all observed combinations indicating the threats included (A=*agriculture*, L=*logging*, E=*exploitation*, I=*intense hab use*, Q=*quality* *loss*, C=*comm disruption*, F=*fragmentation*, and Na=*nature*); the number of species with that combination (*N*) and the percentage (%) they represent from all species with three listed threats. Combinations represented in the main text figure 3 and supplementary figure S2 are in bold.
