# Supplementary material for: Generalized Drivers in the Mammalian Endangerment Process
Source: PLoS One. 2014 Feb 26;9(2):e90292. doi: 10.1371/journal.pone.0090292 (PMC3936011; doi:10.1371/journal.pone.0090292)
Supplement: Table S5 — Observed threat combinations for mammals with four listed threats. (DOCX) [file pone.0090292.s007.docx]

**Table S5.** Observed threat combinations for mammals with four listed threats.

| A | E | L | Q | I | C | F | Na | *N* | % |
| --- | --- | --- | --- | --- | --- | --- | --- | --- | --- |
| **x** | **x** | **x** |  | **x** |  |  |  | **84** | **25.5** |
| **x** | **x** | **x** | **x** |  |  |  |  | **43** | **13.0** |
| x |  | x | x | x |  |  |  | 26 | 7.9 |
| x | x |  |  | x | x |  |  | 14 | 4.2 |
| x |  | x |  | x |  | x |  | 14 | 4.2 |
| x | x |  | x | x |  |  |  | 13 | 3.9 |
| x | x |  |  | x |  |  | x | 12 | 3.6 |
| x |  | x |  | x | x |  |  | 12 | 3.6 |
| x | x | x |  |  | x |  |  | 11 | 3.3 |
| x |  |  | x | x | x |  |  | 10 | 3.0 |
| x |  | x | x |  | x |  |  | 9 | 2.7 |
| x | x | x |  |  |  | x |  | 8 | 2.4 |
|  |  |  | x | x | x |  | x | 8 | 2.4 |
|  | x |  | x | x |  |  | x | 6 | 1.8 |
| x | x |  |  | x |  | x |  | 5 | 1.5 |
|  | x | x | x | x |  |  |  | 5 | 1.5 |
| x | x |  |  |  | x | x |  | 5 | 1.5 |
|  | x |  | x | x | x |  |  | 5 | 1.5 |
|  | x |  | x |  | x | x |  | 4 | 1.2 |
| x |  | x |  | x |  |  | x | 4 | 1.2 |
| x | x |  | x |  |  | x |  | 3 | 0.9 |
|  | x |  |  | x | x |  | x | 3 | 0.9 |
| x |  |  | x | x |  | x |  | 3 | 0.9 |
| x |  |  | x | x |  |  | x | 3 | 0.9 |
|  | x |  | x | x |  | x |  | 2 | 0.6 |
| x | x |  | x |  | x |  |  | 2 | 0.6 |
| x |  |  | x |  | x | x |  | 2 | 0.6 |
|  |  | x |  | x | x |  | x | 2 | 0.6 |
| x |  |  |  | x | x |  | x | 2 | 0.6 |
|  | x | x |  | x |  | x |  | 1 | 0.3 |
|  | x | x |  | x | x |  |  | 1 | 0.3 |
|  | x |  |  | x | x | x |  | 1 | 0.3 |
|  | x |  |  | x |  | x | x | 1 | 0.3 |
| x |  | x | x |  |  | x |  | 1 | 0.3 |
| x |  | x |  |  | x | x |  | 1 | 0.3 |
| x |  |  |  | x | x | x |  | 1 | 0.3 |
|  |  | x | x | x | x |  |  | 1 | 0.3 |
|  |  | x | x | x |  |  | x | 1 | 0.3 |
|  |  |  |  | x | x | x | x | 1 | 0.3 |

We list all observed combinations indicating the threats included (A=*agriculture*, L=*logging*, E=*exploitation*, I=*intense hab use*, Q=*quality* *loss*, C=*comm disruption*, F=*fragmentation*, and Na=*nature*); the number of species with that combination (*N*) and the percentage (%) they represent from all species with four listed threats. Combinations represented in the main text figure 3 and supplementary figure S2 are in bold.
